# An Expanding World of Novel Psychoactive Substances: Opioids

**DOI:** 10.3389/fpsyt.2017.00110

**Published:** 2017-06-30

**Authors:** Jolanta B. Zawilska

**Affiliations:** ^1^Department of Pharmacodynamics, Medical University of Lodz, Lodz, Poland

**Keywords:** novel psychoactive substances, opioids, fentanyls, MT-45, AH-7921, U-47700, toxicity, naloxone

## Abstract

The abuse of novel psychoactive substances (NPS) has been increasing dramatically worldwide since late 2000s. By the end of 2015, more than 560 NPS had been reported to the European Monitoring Centre for Drugs and Drug Addiction. Although the most popular compounds are synthetic cannabinoids and psychostimulatory derivatives of cathinone (so-called β-keto-amphetamines), novel synthetic opioids have recently emerged on the recreational drug market. They include fentanyl (a potent narcotic analgesic) and its analogs (e.g., acetylfentanyl, acryloylfentanyl, carfentanil, α-methylfentanyl, 3-methylfentanyl, furanylfentanyl, 4-fluorobutyrylfentanyl, 4-methoxybutyrylfentanyl, 4-chloroisobutyrylfentanyl, 4-fluoroisobutyrylfentanyl, tetrahydrofuranylfentanyl, cyclopentylfentanyl, and ocfentanil) and compounds with different chemical structures, such as AH-7921, MT-45, and U-47700. This survey provides an overview of the pharmacological properties, pattern of use, and desired and unwanted effects of the above-listed novel opioids. Special emphasis is given to cases of non-fatal and lethal intoxication involving these compounds.

## Introduction

The last decade has seen a worldwide surge in the recreational use of novel psychoactive substances (NPS). Although various products are labeled with warnings “not for human consumption,” they are intended to mimic the psychoactive effects of illicit drugs of abuse. Between 2008 and 2015, a total of 644 NPS were reported by 102 countries to the United Nations Office for Drugs and Crime (1) and by the end of 2015, a total of 561 of NPS had been notified to the European Monitoring Centre for Drugs and Drug Addiction (EMCDDA) (2). While NPS can be purchased online, from head shops or drug dealers, buying drugs through the internet, both from freely accessible websites, and more recently, from the so-called dark web, has become increasingly popular (3, 4).

Novel psychoactive substances are mainly of synthetic origin (e.g., derivatives and analogs of existing controlled drugs and analogs of pharmaceutical products) and comprise different drug classes, including, among others, synthetic cannabinoids, synthetic cathinones, phenethylamines, piperazines, ketamine- and phencyclidine-type substances, tryptamines, benzofurans, and opioids (5). Although the most popular of these have been cannabinoids and designer cathinones, recent years have seen the appearance of novel synthetic opioids on the recreational drug market (1, 2, 6). By analogy to other NPS groups, the primary motivation for using designer opioids is pleasure and enjoyment. In addition, the use of opioids is markedly motivated by habit, addiction, and coping with life challenges (7). Designer opioids pose an especially serious concern for public health as they are endowed with a high potency and are often sold under the guise of heroin to unsuspecting users (8). Many of them are derivatives of therapeutically used drugs, namely fentanyl. However, new synthetic opioids such as AH-7921, MT-45, and U-47700, with structures distinct from those of known therapeutic or recreational drugs, have also emerged. They are used on their own or in combination with other opioids. The aim of the current contribution is to present updated information on the properties of novel synthetic opioids. Special attention is given to the acute toxic effects exerted by this group of NPS.

## Methods

A literature search was performed on two representative databases (PubMed and Google Scholar) and various governmental and institutional websites, using the following keywords alone or in combination: NPS, synthetic opioid, fentanyl, illicit fentanyls, names of particular designer analogs of fentanyl, MT-45, AH-7921, U-47700, toxicity, and naloxone. Only those articles that had abstracts available in the English language were included. All articles were screened from their abstracts to determine their relevance in the framework of the current review.

## Fentanyl, Carfentanil, and Non-Pharmaceutical Fentanyls (NPF)

Fentanyl, *N*-phenyl-*N*-[1-(2-phenylethyl)piperidin-4-yl]propanamide, was first synthesized by Paul Janssen and his research team from Janssen Pharmaceutical (Belgium) in 1960 as an opioid analgesic agent. It was introduced into medical practice as an intravenous anesthetic under the trade name of Sublimaze in 1960s (9). The drug is a potent agonist of μ-opioid receptors, with an activity 50–100 times higher than morphine. Fentanyl quickly crosses the blood–brain barrier due to its high lipid solubility; it has a rapid onset and short duration of action. The drug is used as a narcotic analgesic supplement in general and regional anesthesia as well as in management of severe chronic pain and postoperative pain (9). Fentanyl pharmaceutical products are available in forms of oral transmucosal lozenges (Actiq^®^), buccal tablets (Fentora™), sublingual tablets (Abstral^®^), sublingual spray (Subsys™), nasal spray (Lazanda^®^), transdermal patches (Duragesic^®^ and generics), and injectable formulations. Fentanyl-containing transdermal patches are used to treat patients with chronic pain who require continuous opioid analgesia. The recommended serum concentration is 1–2 ng/mL for analgesia and 10–20 ng/mL for anesthesia (9, 10).

In addition to analgesia, fentanyl and its analogs (hereafter fentanyls) depress the respiratory system, constrict the pupils, and produce drowsiness and euphoria, the latter being less pronounced than with heroin and morphine (9, 10). The most common side effects include nausea, dizziness, vomiting, fatigue, headache, and constipation. Repeated use of fentanyls leads to the development of tolerance and dependence. Characteristic withdrawal symptoms include sweating, anxiety, diarrhea, bone pain, abdominal cramps, and shivers or “goose flesh” (9, 10). Due to the narrow therapeutic index, the use of fentanyls in the recreational drug scene is exceptionally dangerous, especially in opioid intolerant users. High doses might result in death due to respiratory arrest and pulmonary edema. Importantly, serious interactions can occur when fentanyls are mixed with heroin, cocaine, alcohol, and other CNS depressants, in particular benzodiazepines [e.g., Ref. (10, 11)]. The most common therapies administered to patients intoxicated with fentanyls include naloxone, oxygen, intubation, and intravenous fluids (10).

As fentanyl and its analogs are endowed with high abuse liability, dependence potential and toxicity (see below), all fentanyls approved for medical use are internationally controlled, as well as several compounds from this group that have never been developed into pharmaceutical products (12) (Table 1).

**Table 1:** Fentanyl and its analogs controlled under the 1961 Single Convention on Narcotic Drugs (12).

Year	Compound
1964	Fentanyl
1980	Sufentanil
1984	Alfentanil
1988	α-Methyl-thiofentanyl, β-hydroxyfentanyl, β-hydroxy-3-methylfentanyl, 3-methylfentanyl, *para*-fluorofentanyl, thiofentanyl
2016	Acetylfentanyl

The first documented large-scale illicit use of fentanyl (street names: “China White,” “Synthetic Heroin,” “Drop Dead,” “Flatline,” “Lethal Injection,” “Apache,” “China Girl,” “Chinatown,” “Dance Fever,” “Great Bear,” “Poison,” and “Tango & Cash”) was in the USA, mainly in California, between 1979 and 1988 (13–15). Fentanyl exposures reported to the American Association of Poison Control Centers increased from 300 in 2010 to 1,724 in 2011, and since then have remained steadily high (1,632 in 2012, 1,486 in 2013, and 1,418 in 2014) (16). In March 2015, the United States Drug Enforcement Administration (DEA) issued a nationwide warning of fentanyl laced in heroin causing significant health problems across the USA (16). Between 2009 and 2014, there were at least 1,019 drug poisoning deaths in Canada where postmortem toxicological screening indicated the presence of fentanyl. More than half of these deaths occurred in the latter 2 years, 2013 and 2014 (17). In Europe, the first cases of fatal intoxication with fentanyl were reported in Sweden (18). Later on, illicit fentanyl use became a serious health problem in Estonia, with an estimated number of 1,100 deaths during 2005–2013 (19). Outside of Estonia, 180 fentanyl-related deaths were reported in Sweden (2006–2013), 160 in Germany (2007–2011), 70 in the UK (2007–2012), 40 in Finland (2008–2010), and five in Greece (2005–2011) (19). According to epidemiological data, the increase in use of fentanyls in Europe and in the USA was largely associated with the low availability, low purity, and/or high price of heroin, features that were at least partially linked to the imposition of Taliban control on opium production (10, 19).

Among various fentanyl-containing products that are available on prescription, abuse of transdermal fentanyl patches has received increasing attention in recent years. These patches can be misused in a variety of ways: (1) they can be placed in a glass containers, heated, and smoked, (2) gel contents removed from the patches can be injected or ingested, (3) fentanyl in patches can be scratched and smoked, (4) patches are simmered in a small volume of water and the obtained solution is injected intravenously, (5) frozen patches are cut into pieces and then chewed, placed under the tongue, or in the cheek cavity for drug absorption through the oral mucosa or inserted into the rectum [e.g., Ref. (20–31)]. It has been reported that chewing a fentanyl patch could quickly decrease the user’s level of consciousness and result in the intrabronchial aspiration of the patch, a clinical feature that intensifies fentanyl-induced breathing problems (26).

Carfentanil [methyl 1-(2-phenylethyl)-4-(*N*-propanoylanilino)piperidine-4-carboxylate; carfentanil, 4-carbomethoxyfentanyl], a fentanyl analog, was first synthesized by chemists at Janssen Pharmaceutical in 1974 and marked under the brand name Wildnil. The drug is a very potent agonist of opioid receptors, with the rank order of potency: μ >>> δ > κ. Binding studies found the calculated Ki values for human opioid receptors to be 0.024 nM (μ_1_), 3.3 nM (δ), and 43 nM (κ). By comparison, Ki values for fentanyl were 1.9 nM (μ_1_), 153 nM (δ), and 197 nM (κ).^1^ It is estimated that the clinical potency of carfentanil is 10,000 times that of morphine, 4,000 times that of heroin, and 100 times that of fentanyl, making it one of the most potent known and the most potent commercially used opioids. The drug is approved to be used only by veterinarians as a tranquilizing agent for large wildlife animals, such as elephants and bears, for examination and procedures. The first confirmed case of a human being poisoned with carfentanil was published in 2010 (32). A 42-year old veterinarian was accidentally splashed in the eyes and mouth with a dart containing 1.5 mg carfentanil citrate and 50 mg xylazine. Despite immediate decontamination, the man became drowsy within 2 min. The patient was administered 100 mg parenterally of naltrexone and transported to the hospital, where he fully recovered (32). Recently, alarming reports from the USA and Canada show that carfentanil has been increasingly laced with or disguised as heroin. The drug has already been connected to hundreds of overdose cases, many of them fatal [e.g., Ref. (33–37)].

**Figure 1:**
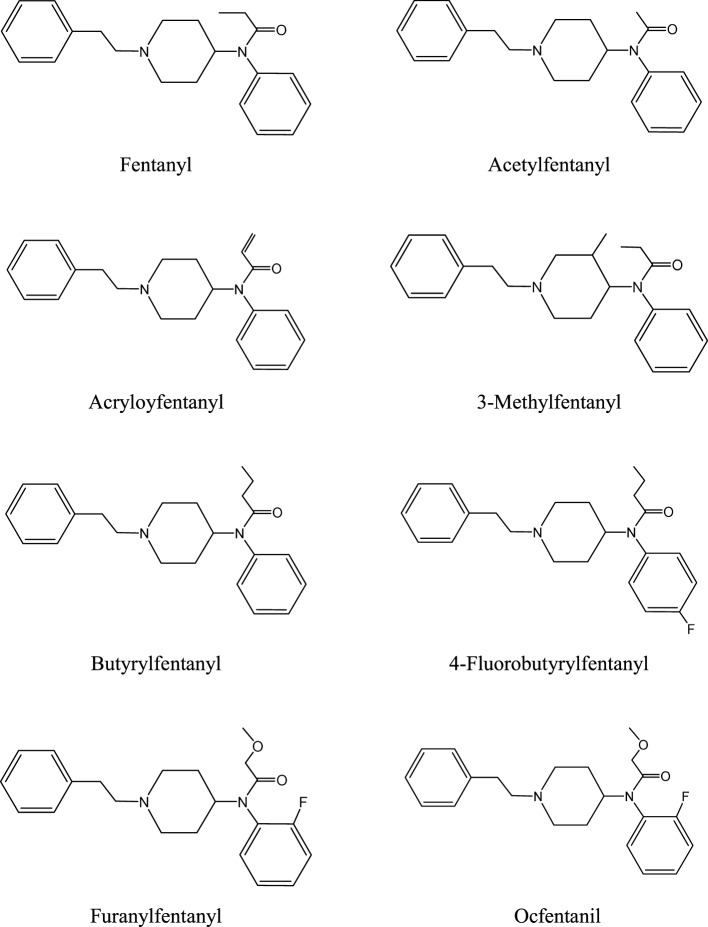
Chemical structures of fentanyl and its analogs.

Several fentanyl analogs are clandestinely synthesized for recreational use (10, 12, 19, 38–40). These compounds have been developed by modification or replacement of the fentanyl’s propionyl chain or by replacement of its ethylphenyl moiety. The obtained analogs have been further modified by substitution with fluoro-, chloro-, or methoxy- groups at the *N*-phenyl ring. Examples of fentanyls that have not been approved for medical use, the so-called NPF, are listed below (see also Figure 1).

- acetylfentanyl (*N*-phenyl-*N*-[1-(2-phenylethyl)piperidin-4-yl]acetamide; acetyl fentanyl, desmethyl fentanyl, MCV 4848, NIH 10485),
- acryloylfentanyl (*N*-phenyl-*N*-[1-(2-phenylethyl)piperidin-4-yl]prop-2-enamide, acrylfentanyl, acryloyl-F, Acr-F, ACF),
- α-methylfentanyl (*N*-phenyl-*N*-[1-(1-phenyl-2-propanyl)piperidin-4-yl]propanamide),
- 3-methylfentanyl (*N*-[3-methyl-1-(2-phenylethyl)piperidin-4-yl]-*N*-phenylpropanamide; mefentanyl, 3-MF),
- butyrylfentanyl (*N*-phenyl-*N*-[1-(2-phenylethyl)piperidin-4-yl]butanamide; butyl fentanyl; BF),
- 4-methoxybutyrylfentanyl (*N*-(4-methoxyphenyl)-*N*-[1-(2-phenylethyl)piperidin-4-yl]butanamide; 4-MeO-BF),
- 4-fluorobutyrylfentanyl (*N*-(4-fluorophenyl)-*N*-[1-(2-phenylethyl)piperidin-4-yl]butanamide; 4-FBF),
- 4-fluoroisobutyrylfentanyl (*N*-(4-fluorophenyl)-2-methyl-*N*-[1-(2-phenylethyl)piperidin-4-yl]propanamide; 4F-iBF),
- 4-chloroisobutyrylfentanyl (*N*-(4-chlororophenyl)-2-methyl-*N*-[1-(2-phenylethyl)piperidin-4-yl]propanamide; 4F-iBF),
- furanylfentanyl (*N*-phenyl-*N*-[1-(2-phenylethyl)piperidin-4-yl]-2-furancarboxamide; furafentanyl),
- cyclopentylfentanyl (*N*-(1-phenylethylpiperidin-4-yl)-*N*-phenylcyclopentanecarboxamide; CP-F),
- tetrahydrofuranylfentanyl (*N*-(1-phenethylpiperidin-4-yl)-*N*-phenyltetrahydrofuran-2-carboxamide; tetrahydrofuranfentanyl, THF-F), and
- ocfentanil (*N*-(2-fluorophenyl)-2-methoxy-*N*-[1-(2-phenylethyl)piperidin-4-yl]acetamide; ocfentanil, A-3217).

The reported doses and duration of action of fentanyls in comparison to morphine and heroin^2^ are presented in Table 2.

**Table 2:** Doses and duration of action of synthetic opioids (see text foonote 2).

Route of administration	Dose			Action		
	Light	Common	Strong	Onset	Duration	After-effects
**Morphine**						
	**5–10 mg**	**15–20 mg**	**>30 mg**			
Insufflation				10–30 min	4–5 h	1–12 h
Intravenous/intramuscular				0–1 min	2–4 h	1–12 h

**Heroin**						
Insufflation	7.5–20 mg	20–35 mg	35–50 mg	10–15 min	3–6 h	1–24 h
Smoked	5–15 mg	15–25 mg		5–10 min	3–5 h	1–24 h
Intravenous		5–10 mg	8–15 mg	0–5 min	4–5 h	1–24 h

**Fentanyl**						
Intranasal	10–25 µg	25–50 µg	50–75 µg			
Transdermal	12.5 µg/h	25–50 µg/h	50–100 µg/h	2–4 h	48–72 h	
Buccal				15–30 min	1–4 h	
Insufflated				15–30 min	1–4 h	

**Acetylfentanyl**						
Oral	1–3 mg	3–5 mg	5–7 mg	Minutes	Hours	1–8 h

**Acryloylfentanyl**						
Insufflation	5–12.5 µg	12.5–25 µg	25–47.5 µg	1–5 min	10–30 min	1–2 h

**Butyrylfentanyl**						
Oral	0.4–0.8 mg	0.8–1.5 mg	1.5–3 mg	15–30 min	3–4 h	1–4 h

**4-Fluorobutyrylfentanyl**						
Insufflation	0.3 mg	0.6–0.9 mg	0.9–1.2 mg	Minutes	30–60 min	

**4-Methoxybutyrylfentanyl**						
Oral				5–15 min	45–120 min	1–2 h
Insufflation				1–2 min	30–75 min	1–2 h

**Furanylfentanyl**						
Oral	0.3–0.5 mg	0.5–0.9 mg	0.9–1.6 mg			
Insufflation	0.2–0.4 mg	0.4–0.8 mg	0.8–1.6 mg	1–10 min	1–3 h	1–3 h

**AH-7921**						
Oral	5–10 mg	10–25 mg	>25 mg	15–45 min	6–8 h	1–6 h

**U-47700**						
	**5–7.5 mg**	**7.5–15 mg**	**15–25 mg**			
Oral				15 min	5–7 h	1–4 h
Insufflation				15 min	3–4 h	1–4 h
Intravenous				0–1 min	1–2 h	1–4 h

**MT-45**						
Oral	30–45 mg	45–60 mg	>60 mg	30–45 min	4–6 h	2–3 h

As in the case of other NPS groups, new designer opioids quickly replace the scheduled ones. For example, following the ban of acryloylfentanyl in 2016, four new fentanyls, i.e., 4-chloroisobutyrylfentanyl, 4-fluoroisobutyrylfentanyl, tetrahydrofuranylfentanyl, and cyclopentylfentanyl, appeared on the Swedish drug market (38).

Commonly, fentanyls are sold as powders, nasal sprays, liquids, or in tablet forms. Clandestine opioids are often up mixed with heroin (“fake heroin”) to masquerade heroin, included in cocaine products or black tar heroin, or pressed into counterfeit prescription pills (12, 40, 41).

The first designer fentanyls were α-methylfentanyl and its more potent and dangerous successor, 3-methylfentanyl; the two substances which appeared on the illicit drug market in California in 1978 and 1984, respectively, laced in heroin products or as a heroin substitute [reviewed in Ref. (19)]. The slang terms for these two fentanyls include “China white,” “China girl,” “Persian white,” “egg white,” “crocodile,” and “synthetic heroin.” Importantly, 3-methylfentanyl is one of the most potent opioids that has been widely sold on the black market; its *cis*-(+)-isomer is approximately 7,000 times more potent than morphine, and the *trans*-(±)-isomer is about 1,000 times as potent (42). Numerous fatalities involving 3-methylfentanyl were reported in Estonia in the period 2004 to 2008. In the majority of cases, fentanyl was detected in blood samples together with 3-methylfentanyl [e.g., Ref. (43, 44)].

Three other illicit compounds from this class, acetylfentanyl, butyrylfentanyl, and 4-fluorobutyrylfentanyl, were first notified by the European Early Warning System in 2014. They were typically seized in powder form or tablet form, and, to a lower extent, in liquids and in capsules (31, 45, 46). As acetylfentanyl is often the first synthetic opioid used by those who want to try new opioids, it is colloquially called “the first Apostle of extinction” (47). Acetylfentanyl is five to 15 times more potent than heroin, 80 times more potent than morphine and 15 times less active than fentanyl (45, 48, 49), whereas the potency of butyrylfentanyl was found to be seven times higher than that of morphine and 13 times lower than fentanyl (46, 49, 50). Acetylfentanyl, butyrylfentanyl, and 4-fluorobutyrylfentanyl are typically administered orally, nasally (using sprays), by snorting, smoking, and by intravenous injection (19, 45, 46). By 2014, Germany, Poland, Sweden, and the UK had reported eight acute intoxications and 32 deaths associated with acetylfentanyl to EMCDDA (45). Furthermore, since 2012, acetylfentanyl has been found to be involved in at least 12 deaths in Russia, three in Japan, and more than 50 in the USA (51–59). From April to November 2015, 14 analytically confirmed intoxications with fentanyls (including one fatal) were reported to the Swedish STRIDA project (59). The concentrations of drugs in biological samples were as follows: acetylfentanyl (*n* = 8)—serum 0.6–51.6 (mean 18.3) ng/mL, urine 2.4–3,180 (mean 939) ng/mL; 4-methoxybutyrylfentanyl (*n* = 3)—serum 1.3–11 (mean 5.1) ng/mL, urine 15.8–1,000 (mean 348) ng/mL; and furanylfentanyl (*n* = 2)—serum 4.4 and 148 ng/mL, urine 1,779 and 1,430 ng/mL. Most of the cases were also positive for not only other drugs, mainly benzodiazepines, but also synthetic cathinones, amphetamine, cocaine, and other opioids (oxycodone, tramadol, and fentanyl) (59). Case reports of fatal intoxication involving acetylfentanyl are summarized in Table 3.

**Table 3:** Case reports of fatalities involving novel synthetic opioids.

Gender/age	Case data	Toxicological findings	Ref.
**Acetylfentanyl**			
M/32	A deceased was found dead in the bed in a supine position. Snorting at least 12 h before death. Insufflation straws were found in his bag and in the drawers of a chest. At autopsy: pulmonary edema with mild to severe intraalveolar hemorrhage.	Acetylfentanyl was detected in heart blood, urine and gastric contents.	(52)
M/early 30s	A deceased was found at home, not breathing. A small plastic bag with a pale brown white powder and a syringe with a small amount of liquid were found at the scene. Acetylfentanyl and 4-methoxy-PV8 were detected in both the powder and the liquid. At autopsy: congested lungs, petechiae on eyelid conjunctiva, capsula cordis and pleura, fluidity of the heart blood, and two very recent forearm needle marks. History of habitual “bath salt” use.	Acetylfentanyl: femoral blood, 153 ng/mL; urine, 240 ng/mL; gastric contents, 880 ng/mL. 4-MethoxyPV8: femoral blood, 389 ng/mL; urine, 245 ng/mL; gastric contents, 500 ng/mL. Additionally in femoral blood: 7-aminonitrazepam (200 ng/mL), phenobarbital (7,700 ng/mL), methylphenidate (30 ng/mL), chlorpromazine, and risperidone.	(53)
M/24	A deceased was found unresponsive with uncapped syringe and rubber tourniquet. At autopsy: pulmonary congestion and edema, three recent punctures in left forearm. History of heroin abuse, with two previous overdoses.	Acetylfentanyl: peripheral blood, 260 ng/mL; heart blood, 250 ng/mL; vitreous humor, 240 ng/mL; urine, 2,600 ng/mL.	(55)
M/28	A deceased was found in the bathroom with a tourniquet secured around his arm and a syringe nearby. At autopsy: marked pulmonary and cerebral edema and needle track marks. History of illicit drug abuse.	Acetylfentanyl: subclavian blood, 235 ng/mL; vitreous humor, 131 ng/mL; urine, 234 ng/mL; liver, 2,400 ng/g.	(57)
M/20	A deceased was found dead at home. History of illicit drug abuse.	Acetylfentanyl: heart blood, 285 ng/mL; femoral blood, 192 ng/mL; urine, 3,420 ng/mL; liver, 1,100 ng/g; brain, 620 ng/g. Additionally in heart blood: methoxetamine and fluoxetine.	(58)
F/50	A deceased was found unresponsive in bed. History of bilateral knee replacement, chronic pain, depression and seizures, prescription drug abuse, and ethanol abuse.	Acetylfentanyl: heart blood, 219 ng/mL; femoral blood, 255 ng/mL; urine, 2,720 ng/mL. Additionally in heart blood: venfalaxine, nordiazepam, and chlordiazepoxide.	(58)
**Butyrylfentanyl**			
M/23	A deceased was found unresponsive in the bathroom. A tray with traces of white powder and a tube were found in the bedroom. At autopsy: cerebral edema and small amounts of residual white powder in the nose. History of drug use.	Butyrylfentanyl: peripheral blood, 66 ng/mL; heart blood, 39 ng/mL; liver, 57 ng/g; kidney, 160 ng/g, muscle, 100 ng/g.	(60)
F/53	A deceased was found unresponsive in the bathroom. At autopsy: edematous and congested lungs. History of smoking, prescription drug abuse, and psychiatric disorder hospitalization.	Butyrylfentanyl: peripheral blood, 99 ng/mL; heart blood, 220 ng/mL; vitreous humor, 32 ng/mL; bile, 260 ng/mL; urine, 64 ng/mL; gastric contents, 590 ng/mL; brain, 93 ng/g; liver, 41 ng/g.	(61)
**Butyrylfentanyl and acetylfentanyl**			
F/49	A deceased was found unresponsive and not breathing on the bed. At autopsy: edematous and congested lungs. History of anxiety, bipolar disorder, and two previous suicide attempts.	Acetylfentanyl: peripheral blood, 21 ng/mL; heart blood, 95 ng/mL; vitreous humor, 68 ng/mL; bile, 330 ng/mL; urine, 8 ng/mL; gastric contents, 28,000 ng/mL; brain, 200 ng/g; liver, 160 ng/g. Butyrylfentanyl: peripheral blood, 3.7 ng/mL; heart blood, 9.2 ng/mL; vitreous humor, 9.8 ng/mL; bile, 49 ng/mL; urine, 2 ng/mL; gastric contents, 4,000 ng/mL; brain, 63 ng/g; liver, 39 ng/g Additionally in peripheral blood: alprazolam, 40 ng/mL and ethanol, 0.11 g/dL.	(61)
M/44	A deceased was found unresponsive on the bathroom floor. A box with drug paraphernalia (used syringes, aluminum foil with black residue, scissors, and alcohol wipes) was found elsewhere. At autopsy: pulmonary edema and congestion, evidence of subacute and chronic intravenous drug use in the antecubital fosse, forearms, left wrist, and ankles. History of heroin use.	Butyrylfentanyl: peripheral blood, 58 ng/mL; heart blood, 97 ng/mL; vitreous humor, 40 ng/mL; urine 670 ng/mL; gastric contents, 170 mg/mL; liver, 320 ng/g. Acetylfentanyl: peripheral blood, 38 ng/mL; heart blood, 32 ng/mL; vitreous humor, 38 ng/mL; urine, 690 ng/mL; gastric contents, <170 mg/mL; liver, 110 ng/g.	(62)
**4-Fluorobutyrylfentanyl**			
M/26	A deceased was found dead at home. History of drug abuse.	4-Fluorobutyrylfentanyl: blood, 91 ng/mL; urine, 200 ng/mL; liver, 902 ng/g; kidney, 136 ng/g.	(63)
F/25	A deceased was found dead at home. History of occasional drug and novel psychoactive substances use.	4-Fluorobutyrylfentanyl: blood, 112 ng/mL; urine, 414 ng/mL; liver, 411 ng/g; kidney, 197 ng/g.	(63)
**Furanylfentanyl**			
M/26	A deceased was found dead in the bathroom. A tourniquet was found around his arm and a used needle next to the body. At autopsy: brain edema and pulmonary edema. History of drug abuse.	Blood (ng/mL): furanylfentanyl, 1.05; Δ^9^-tetrahydrocannabinol (THC), 0.63; mirtazapine, 74.1; desmethylnitrazapine, 31.7; pregabalin, 6,032; buprenorphine, 2.01; norbuprenorphine, 2.86; clonazepam, 21.1; 7-aminoclonazepam, 624. Urine (ng/mL): buprenorphine, 30; norbuprenorphine, 180.	(64)
M/36	A deceased was found lying on the floor of the bathroom. At autopsy: pulmonary edema and froth in the airways. History of drug abuse.	Blood (ng/mL): furanylfentanyl, 7.66; pregabalin, 14,815.	(64)
M/37	A deceased was found lying in the ditch, with a body temperature of 25°C. An empty strip of zopiclone was found nearby. Resuscitation for 35 min was unsuccessful. At autopsy: generalized visceral congestion.	Blood (ng/mL): furanylfentanyl, 0.95; carbamazepine, 9,524; venlafaxine, 9,480; alimemazine, 317; promethazine, 63.5; desmethylpromethazine, 106; methyphenidate, 28.6; ritalinic acid, 762; acetaminophen, 8,466; pregabalin, 33,862; amphetamine, 116; 7-aminoclonazepam, 95.	(64)
M/26	A deceased was found dead on the couch. A used needle, a spoon, and a suspected drug were found at the scene. At autopsy: brain edema and pulmonary edema. History of drug abuse.	Blood (ng/mL): furanylfentanyl, 0.43.	(64)
M/26	A deceased was found dead in his apartment. Three nasal sprays suspected to contain fentanyl were found at the scene. At autopsy: pulmonary edema and froth in the airways. History of drug abuse.	Blood (ng/mL): furanylfentanyl, 0.78; carbamazepine, 14,815; pregabalin, 28,481; gabapentin, 94,937; norbuprenorphine, 1.37; fentanyl, 0.4; alprazolam, 42.2; alimemazine, 211; desmethylalimemazine, 211; diazepam, 31.6; methylphenidate, 4.2; ritalinic acid, 232. Urine (ng/mL): buprenorphine, 6; norbuprenorphine, 30.	(64)
M/27	A deceased was found dead in an apartment shared by drug abusers. History of suicide attempts.	Blood (ng/mL): furanylfentanyl, 1.16.	(64)
M/24	A deceased was found dead on the couch. Drug paraphernalia were found nearby. At autopsy: congested and edematous lungs. History of drug abuse and recent treatment in an addiction center.	Blood (ng/mL): furanylfentanyl, 0.4; fentanyl, 1.27. Urine (ng/mL): fentanyl, 150.	(64)
**Ocfentanil**			
M/16	A deceased was found dead at home, seated and leaning forward on the toilet. Drug paraphernalia, brown powder in a small zip-locked plastic bag lying on a card with a straw were found at the scene. History of illicit drug abuse and depression.	Ocfentanil: femoral blood, 15.3 ng/mL; heart blood, 23.3 ng/mL; vitreous humor, 12.5 ng/mL; urine 6.0 ng/mL; bile, 13.7 ng/mL; liver, 31.2 ng/g; kidney, 51.2 ng/g; brain, 37.9 ng/g; nose mucus membrane, 2,999 ng/swab. Additionally in peripheral blood: acetaminophen, 45 µg/mL; caffeine, 230 ng/mL.	(65)
M/24	A deceased was found dead in his apartment. Drug paraphernalia, plastic zipper bag with brown powder, identified as ocfentanil, were found at the scene. At autopsy: lung congestion and edema, brain congestion and edema. History of illicit drug use.	Ocfentanil: peripheral blood, 9.1 ng/mL; heart blood, 27.9 ng/mL; urine, 480 ng/mL. Additionally in peripheral blood: citalopram (130 ng/mL); quetiapine (<10 ng/mL), THC (2.8 ng/mL), and carboxy-THC (<5 ng/mL).	(66)
**AH-7921**			
M/early 20 s	A deceased, victim of a minor traffic accident, was discharged from hospital the following day with a prescription for 30 mg codeine/400 mg acetaminophen. He ingested six tablets and some powder from zip-lock bags marked 3-methylmetcathinon (3-MMC) and 4-fluoromethamphetamine (4-FMA) bought on the internet. Soon after ingestion, when lying on the floor, he began to snore and was unresponsive. At autopsy: pulmonary edema.	Peripheral blood: AH-7921, 430 ng/mL; 2-FMA, 6.9 ng/mL; 3-MMC, 2.1 ng/mL; codeine, 420 ng/mL; acetaminophen, 18,700 ng/mL.	(67)
F/young	A deceased was found dead at home. Used needles and small plastic bags labeled “AH-7921” and “etizolam” were found in waste bins. At autopsy: needle marks in various stages of healing on the right cubital fossa.	Peripheral blood: AH-7921, 330 ng/mL; methoxetamine, 64 ng/mL; etizolam, 270 ng/mL; phenazepam, 1,330 ng/mL; 7-aminonitrazepam, 43 ng/mL; diazepam, 46 ng/mL; oxazepam, 18 ng/mL; nordiazepam 73 ng/mL.	(67)
M/19	A deceased was found dead on the bed. Frosty substance around the mouth. At autopsy: pulmonary congestion and edema.	AH-7921: peripheral blood, 6,600 ng/mL; heart blood, 3,900 ng/mL; urine, 6,000 ng/mL; bile, 17,000 ng/mL, liver, 26,000 ng/g; kidney, 7,200 ng/g; brain, 7,700 ng/g.	(68)
F/22	A deceased was found dead in the bedroom of her apartment. A plastic bag labeled “AH-7921” was found in the apartment. At autopsy: cerebral edema with increased intracranial pressure, the internal organs full of blood. History of drug abuse and AH-7921 use.	AH-7921: femoral blood, 450 ng/mL; heart blood, 480 ng/mL; urine, 760 ng/mL; vitreous humor, 190 ng/mL; stomach content, 40 µg/mL; liver, 530 ng/g.	(69)
**U-47700**			
M/20	A deceased was found dead with a syringe clutched in his hand. Drug paraphernalia were located to his proximity. History of drug abuse.	Blood: U-47700, 382 ng/mL; amphetamine, 12 ng/mL.	(70)
M/39	A deceased was found unresponsive lying on the sofa; a syringe was found on the floor. History of ordering designer drugs on the internet.	Blood: U-47700, 217 ng/mL; mephedrone, 22 ng/mL.	(70)
M/25	A deceased was found unresponsive with symptoms of pulmonary edema. A white powder, determined to be U-47700, was found at the scene. History of polydrug abuse.	Blood: U-47700, 334 ng/mL.	(70)
M/23	A deceased was found on the bathroom floor with a ligature around his arm. Syringe and a pocket containing a powdery substance labeled “U-47700” were found at the scene. History of drug abuse.	Blood: U-47700, 252 ng/mL; citalopram, 43 ng/mL.	(70)
M/29	A deceased was complaining of a headache the day of his death and suddenly collapsed. At the autopsy: pulmonary edema and brain edema.	Blood: U-47700, 453 ng/mL.	(70)
M/29	A deceased was found unresponsive with the evidence of pulmonary edema. A rolled-up 10 dollar bill with a residue of white powder and series of packets with white powder were found at the scene. History of drug abuse.	Blood: U-47700, 242 ng/mL; carboxy-THC, 5.3 ng/mL.	(70)
M/26	A deceased was found dead at home. Five syringes, benadryl and etizolam pills, diphenhydramine tables, and three glass dropper bottles were found at the scene. History of drug abuse.	Blood: U-47700, 103 ng/mL; diphenhydramine, 694 ng/mL.	(70)
M/21	A deceased was found dead at home with an injection site in the right arm containing a needle. History of drug abuse.	Blood: U-47700, 299 ng/mL; tramadol < 250 ng/mL; alprazolam, 47 ng/mL; lorazepam, 11 ng/mL; 3-methoxyphencyclidine, 180 ng/mL.	(70)
M/24	A deceased was found unconscious and unresponsive at home. History of “U-47700” abuse.	Blood: U-47700, 487 ng/mL; etizolam, 86 ng/mL; chlorpheniramine < 250 ng/mL; diphenhydramine, 250 ng/mL.	(70)
M/23	A deceased was found dead sitting up in a chair.	Blood: U-47700, 311 ng/mL; oxycodone, 11 ng/mL; venlafaxine, 2,600 ng/mL; *O*-desmethylvenlafaxine, 380 ng/mL.	(70)
M/24	A deceased was found unresponsive with a syringe in his arm. History of drug abuse.	Blood: U-47700, 59 ng/mL.	(70)
M/46	A deceased was snorting a compound from an envelope labeled “U-47700.” At autopsy: pulmonary congestion and edema.	Peripheral blood: U-4770, 190 ng/mL; alprazolam, 120 ng/mL; doxylamine, 300 ng/mL; diphenhydramine, 140 ng/mL; carboxy-THC, 2.4 ng/mL. Urine: U-47700, 360 ng/mL. Liver: U-47700, 1,700 ng/g.	(71)
Not provided	A deceased was found on the bed. At autopsy: pulmonary congestion.	U-47700: femoral blood, 525 ng/mL; heart blood, 1,347 ng/mL; urine, 1,393 ng/mL; kidney, 270 ng/g; liver, 430 ng/g; lung, 320 ng/g; brain, 97 ng/g. Additionally in blood: diphenidine (ca. 1.7 ng/mL); methoxphenidine (ca. 26 ng/mL); ibuprofen (ca. 1.8 µg/mL); and naloxone (1.9 ng/mL).	(72)
Not provided	A deceased was found on the bed. At autopsy: pulmonary congestion.	U-47700: femoral blood, 819 ng/mL; heart blood, 1,043 ng/mL; urine, 1,848 ng/mL; kidney, 140 ng/g; liver, 3,100 ng/g; lung, 240 ng/g; brain, 110 ng/g. Additionally in blood: diphenhydramine (ca. 45 ng/mL) and methylphenidate (ca. 2.5 ng/mL).	(72)
**U-47700 and furanylfentanyl or fentanyl or butyrylfentanyl**			
M/36	A deceased was found unresponsive in the bathroom with a syringe cup in his mouth. History of drug abuse.	Blood: U-47700, 135 ng/mL; furanylfentanyl, 26 ng/mL.	(70)
M/33	History of heroin and cocaine abuse.	Blood: U-47700, 167 ng/mL; furanylfentanyl, 56 ng/mL; morphine, 48 ng/mL.	(70)
M/29	A deceased was found unresponsive.	Blood: U-47700, 490 ng/mL; furanylfentanyl, 76 ng/mL.	(70)
M/40	History of heroin/opioid abuse.	Blood: U-47700, 105 ng/mL; furanylfentanyl, 2.5 ng/mL.	(70)
M/36	At autopsy: pulmonary edema. History of drug abuse and experimentation with substances purchased over the internet.	Blood: U-47700, 13.8 ng/mL; fentanyl, 10.9 ng/mL. Urine: U-47700, 71 ng/mL.	(73)
M/18	A deceased was found unresponsive in the bed. Syringes and two white powders, determined to be butyrylfentanyl and U-47700, were found at the scene. History of drug abuse.	Blood: U-47700, 17 ng/mL; butyrylfentanyl, 26 ng/mL; ethanol, 0.03 g/dL.	(70)
**MT-45**			
M/24	A deceased was found dead sitting on the chair in front of the desk. An e-cigarette with unknown fluid, drug paraphernalia, and several bags of white powder labeled “Methoxphenidine,” “Methoxmetamine,” and “MT-45” were found at the scene. At autopsy: brain edema, hemorrhagic pulmonary edema, and hyperemia of the internal organs. History of amphetamine abuse.	MT-45: femoral blood, 660 ng/mL; heart blood, 1,300 ng/mL; urine, 370 ng/mL; vitreous humor, 260 ng/mL; gastric content, 49 µg/mL; liver, 24 µg/g. Also in femoral blood: lidocaine and two synthetic cannabinoids—PB-22 and 5 F-APINACA.	(69)
M/35	A deceased was found dead at home. Drug paraphernalia (scale, spoon, pipe, and lighter) and two packets of white powder, one testing positive for MT-45 and the other for etizolam, were found at home. At autopsy: pulmonary congestion and edema. History of substance abuse.	Peripheral blood: MT-45, 520 ng/mL; etizolam, 35 ng/mL; diphenhydramine, 220 ng/mL	(74)

Cole and coworkers present a history of an 18-year-old heroin abuser who had overdosed butyrylfentanyl (75). The man was found unconscious with labored breathing and taken to the emergency department (ED), where he was treated intravenously with 0.4 mg of naloxone. The patient developed a pulmonary edema, acute lung injury, and diffuse alveolar hemorrhage. The man stated that he had snorted what he believed to be acetylfentanyl, which he had purchased over the internet (75). In 2015, the DEA reported 40 confirmed fatalities associated with butyrylfentanyl from three states: Maryland (one), New York (37), and Oregon (one) (50). Other documented and published cases of fatal butyrylfentanyl overdose (60–62) are presented in Table 3. Two analytically confirmed cases of fatal intoxication with 4-fluorobutyrylfentanyl were recently reported in Poland by Rojkiewicz and coworkers (63) (see Table 3).

The first report of a furanylfentanyl-induced intoxication was recorded in December 2015 in the USA. From December 2015 through September 2016, a total of 494 forensic cases of furanylfentanyl, including 128 confirmed fatalities, were reported to the DEA (76). During a 4-day period (July 15–18, 2016), 43 patients in British Columbia, Canada, were diagnosed as intoxicated with crack cocaine contaminated with furanylfentanyl. Most of them were men, with an average age of 42 years (range 18–63) (77). A series of furanylfentanyl-related deaths that occurred in Sweden between 2015 and 2016 (64) are summarized in Table 3.

Ocfentanil was developed in early 1990s in an attempt to obtain an analgesic with less cardiovascular and respiratory side effects than morphine, but it has never been approved for medical use. The drug has recently been detected in the hidden market as an adulterant of heroin (78). Deaths involving ocfentanil were reported in Belgium (65) and in Switzerland (66), and the cases are summarized in Table 3.

Another new synthetic analog of fentanyl, i.e., acryloylfentanyl, has been recently identified in a few European countries: Denmark, Estonia, Finland, Latvia, and Sweden (12, 39). The compound has been typically seized in liquid or in a tablet form and less frequently as a powder or in capsule form (12, 79). Acryloylfentanyl was the most common fentanyl derivative in Sweden from the end of January 2016 (a time when 4-methoxybutyrylfentanyl and furanylfentanyl were banned) to September 2016, after which time it became scheduled as narcotic (12, 38, 39). Limited data indicate that the drug is taken nasally (using a nasal solution or by snorting), orally, and by intravenous injection (12, 39). Twenty-one acute intoxications associated with acryloylfentanyl were reported by Sweden to the EMCDDA; all of them occurred between March and August 2016. In the analytically confirmed cases, the concentration of acryloylfentanyl in serum (*n* = 8) ranged from 0.5 to 2.1 (mean 1.0) ng/mL, and in urine (*n* = 9) from 1.8 to 196 (mean 63) ng/mL (38). Clinical symptoms were generally consistent with the opioid toxidrome. They predominantly included unconsciousness, respiratory depression, and miosis, and less commonly, tachycardia, vomiting/nausea, restlessness/anxiety, low oxygen saturation, dizziness, hypertension, chest pain, cyanosis, blurred vision, constipation, somnolence, hallucinations, and high body temperature (12, 38, 39). Forty-two deaths associated with acryloylfentanyl have been reported in Europe: one in Denmark, one in Finland, one in Latvia, and 39 in Sweden, all of them occurred between April and September 2016. The presence of acryloylfentanyl in biological samples taken from the deceased was confirmed in 40 cases. While acrylyolfentanyl was the only substance detected in two fatal cases, samples from the remaining cases included benzodiazepines and their metabolites, ethanol, antidepressants, antipsychotics, “Z”-drugs, pregabalin, and, to a lesser extent, Δ^9^-tetrahydrocannabinol (THC), synthetic cathinones, synthetic cannabinoids, amphetamine, methylenedioxymethamphetamine (ecstasy), gabapentin, and opioids (12, 39).

## New Generation of Synthetic Opioids: AH-7921, U-47700, and MT-45

**Figure 2:**
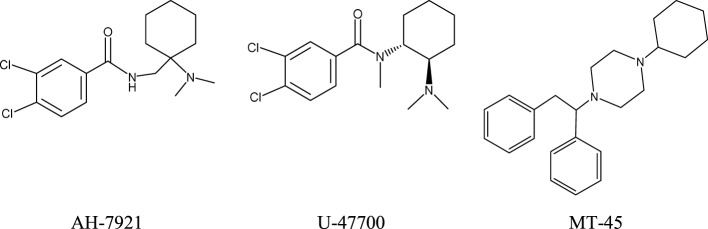
Chemical structures of novel synthetic opioids.

Since 2010, new potent synthetic opioids with chemical structures different from fentanyl, i.e., AH-7921, U-47700, and MT-45, have appeared on the recreational drug market. Their chemical structures are presented in Figure 2. Based on user reports and clinical data, the desired and adverse effects of these compounds resemble those of classical opioids. The reported doses and duration of action of these drugs are presented in Table 2.

### AH-7921

AH-7921, 3,4-dichloro-*N*-{[1-(dimethylamino)cyclohexyl]methyl}benzamide, was invented in mid-1970s by researchers from Aston University in Birmingham (UK) and the pharmaceutical company Allen & Hanburys Ltd. as a potent opioid analgesic agent. However, due to its abuse potential and toxicity AH-7921 has never been developed into a medicine. The compound acts as an agonist of μ-opioid receptors, although at high doses it can also stimulate κ-opioid receptors. In animal studies, AH-7921 produced typical morphine-like actions, i.e., antinociception, respiratory depression, sedation, miosis, inhibition of gut propulsion, and lowered body temperature, with a potency almost equipotent to that of morphine (80, 81).

AH-7921 was first identified in Europe in a sample purchased from an internet retailer in July 2012 (81). The following year, the compound was found in Japan in “legal highs” products containing synthetic cannabinoids and cathinones (82). AH-7921 is sold as a free base and as a hydrochloride salt in a white/off-white powder form (81). It should be emphasized that AH-7921 is also sold or discussed on user websites and public media under the alternative name *Doxylam*. This name could be easily confused with that of doxylamine, a popular antihistamine drug with sedative–hypnotic properties that is present in several over-the-counter medicines; the unintentional use of AH-7921/doxylam for the treatment of allergy or as a hypnotic might have serious health consequences (81).

There is limited information available on the routes of administration and the doses of AH-7921 used. The compound is taken orally, nasally, by smoking, and, less commonly, by intravenous injection (81). By 2014, six non-fatal intoxications associated with AH-7921 had been reported by Sweden to the EMCDDA; five of these were analytically confirmed (81). The clinical symptoms included tachycardia, hypertension, and seizures. The first death associated with AH-7921 use was reported by Norway in December 2012 (81). The next year, a total of 16 cases of fatal intoxications involving AH-7921 were reported by Sweden (10), UK (three), Norway (two), and USA (one). The AH-7921 concentrations in these postmortem blood samples were found to be in the range from 0.03 to 0.99 µg/g (Sweden), 0.05, 0.58, and 4.46 mg/L (UK), 0.33 and 0.43 mg/L (Norway), and 9.1 mg/L (USA) (67–69, 81, 83). In most cases, other psychoactive compounds, mainly benzodiazepines but also amphetamines, synthetic cathinones, codeine, acetaminophen, and methoxetamine, were also detected (67–69, 81, 83). The case reports of fatalities involving AH-7421 are presented in Table 3.

### U-47700

U-47700, 3,4-dichloro-*N*-[2-(dimethyloamino)cyclohexyl]-*N*-methylbenzamide; “Fake morphine,” “U4,” a structural isomer of AH-7921, is a selective μ-opioid receptor agonist developed in 1970s by the chemist Jacob Szmuszkovicz from the Upjohn Company in a search for non-addicting analgesics (84). U-47700 produces morphine-like effects in animals (85). Preclinical studies found U-47700 to be approximately 7.5 times more potent than morphine and about 10 times less potent than fentanyl (85). The compound has not been studied in humans and no pharmacokinetic data exist.

U-47700 was first identified in Sweden in October 2014, and then found in seized powders, tablets, and liquids in various European countries and in the USA (86). The compound is gaining popularity on drug user forums as a legal alternative to morphine/heroin and is typically sold as a white powder. There is limited information available on the routes of administration and the doses of U-47700 used. It is taken orally, nasally, intrarectally, by smoking, intravenous injection, or by combinations of these routes. According to user reports, U-47700 acts longer than AH-7921 (see Table 2).

During 2016, a significant number of U-47700 acute intoxication cases were reported in the USA. Clinical symptoms included respiratory depression, cyanosis, miosis, depressed level of consciousness, drowsiness, tachycardia, nausea, anxiety, and abdominal pain. In most cases, the symptoms were reversed by intravenous injection of naloxone. A 22-year-old man with a history of heroin abuse was found unconscious, apneic, and cyanotic, respiring at four breaths per minute, with a blood pressure of 138/88 mmHg and a pulse of 134 per minute. He was comatose with a Glasgow Coma Scale (GCS) of 3 (87). After recovery by an injection of naloxone, the patient reported using the opioid agonist U-47700, which he had acquired over the internet for recreational purposes (87). A 23-year-old woman insufflated and injected a drug called “U4” and within minutes became unresponsive (88). Paramedics found her cyanotic, respiring at four breaths per minute and with an oxygen saturation percentage in the 60s. Her chest X-ray revealed mild congestion consistent with pulmonary edema. Toxicological analysis of her serum and urine samples detected U-47700 at concentrations of 228 and 393 ng/mL, respectively. In addition, one U-477000 metabolite was found in her serum and four in her urine (88). A 41-year old woman presented to ED with pinpoint pupils for a depressed level of consciousness (89). The patient reported that she had ingested three tablets of what she believed to be Norco, which had been illicitly purchased, to relieve chronic back pain. Toxicological analysis of serum samples identified the following compounds (in ng/mL): acetaminophen, 10,033; benzoylecgonine, 46.6; fentanyl, 15.2; gabapentin, 351; hydrocodone, 107.7; sertraline, 15.7; and U-47700, 7.6 (89). A 29-year-old man was found unresponsive after intravenous injection of U-47700. He spontaneously regained consciousness. Concentrations of U-47700 and phenazepam in serum samples were 240 ng/mL and 1.4 mg/mL, respectively (90). Domanski and coworkers (91) described the case of a 26-year-old man and 24-year-old woman who consumed alcohol and insufflated a powdered substance named U-47700 purchased on the internet that they believed to be a “synthetic cocaine.” Approximately 3 h after use, the man was found with agonal breathing; he was cyanotic, with oxygen saturation of 50%, GCS of 3, and pinpoint pupils. The chest X-ray showed bilateral pulmonary consolidation. At the hospital, the patient was sedated with propofol. The female partner reported that after insufflation of U-47700 she had been feeling “cool and relaxed,” then had fallen asleep and awoken about 3 h later with symptoms of anxiety, nausea, drowsiness, and abdominal pain. Urine samples of both patients were positive for U-47700 (91). Four cases of acute intoxication with U-47700 were reported by Fleming and coworkers (92). One patient presented to ED with cyanosis, miosis, CNS and respiratory depression, and sinus tachycardia. Another one was euphoric, but suffered from nausea, anxiety, abdominal pain, and shivering. Both patients believed they had purchased “synthetic cocaine” from the internet and insufflated the powder substance. Toxicological analysis revealed the presence of U-47700 (urine, case one) and ethanol in blood samples of both patients (92). In the third case, a patient went into cardiac arrest and was administered naloxone. U-47700 was detected in his urine at a concentration of 224 ng/mL (92). In the last case, U-47700 (140 ng/mL) was found in a patient’s urine sample (92). The case reports of analytically confirmed deaths involving U-47700 (70–73, 93) are summarized in Table 3.

### MT-45

MT-45, 1-cyclohexyl-4-(1,2-diphenylethyl)piperazine (also known as IC-6, CDEP, and NSC 299236), was developed in 1970s by Dainippon Pharmaceuticals Co. in Japan as an alternative to morphine for analgesia (94). The free amine of MT-45 is a colorless solid, while the dihydrochloride salt of MT-45 is an off-white solid. MT-45 is usually sold as a white or off-white powder. The compound exists in two enantiomer forms. Racemic MT-45 and the *S*-MT-45 enantiomer exert opioid-like analgesic effects in animals, with the *S*-MT-45 being more potent than morphine. Data from studies performed on mice suggest that MT-45 may have a dependence potential in humans. The pharmacological activity of MT-45 is complex and involves stimulation of δ- and κ-opioid receptors, but also includes interactions with non-opioid molecular targets that are currently not fully understood (95, 96).

There is limited information available on the routes of administration and the doses of MT-45 used. The compound is typically administered orally or by nasal insufflations, although snorting MT-45 causes an intolerable level of irritation in some users, and inhalation, while intravenous or intramuscular injection and rectal insertion are less common (95, 96). The tentative single doses and durations of MT-45 action as reported by users are presented in Table 2.

MT-45 was first reported to the EMCDDA by Sweden in December 2013. The next year, Helander and coworkers published data from nine non-fatal intoxication cases associated with MT-45 that had been reported from November 2013 to February 2014 within the Swedish STRIDA project (97). All patients were men aged 17–32 years. In four cases, MT-45 was the sole compound identified in blood and urine samples, while one or several psychoactive substances (carboxy-THC, pyrazolam, flubromazepam, dextromethorphan, methiopropamine, 3-methoxyphencyclidine, and 3-methylmethcathinone) were detected together with MT-45 in the urine of five patients. The MT-45 concentration in blood was in the range from 6 to 157 (mean 60) ng/mL (97). The majority of patients presented clinical symptoms of opioid intoxication: a decreased level of consciousness or coma (seven cases), respiratory depression or cyanosis (seven cases), and miosis (three cases). Neurological disturbances such as paresthesia in the hands and feet, hand weakness, balance disturbances, vision impairments, and hearing impairment or loss were reported in four cases (97). Unusual symptoms of intoxication that probably involved MT-45 were observed in three Swedish men aged 23–34 years: loss and depigmentation of hair that was most apparent on the eyelashes and eyebrows, widespread folliculitis and dermatitis, painful intertriginous dermatitis, and elevated liver enzymes (98). Two of the men also had lines of discoloration across the nails of the fingers and toes. One patient reported loss of smell and taste. Two patients suffered from tremors and coldness for months. The symptoms gradually disappeared over time. Notably, in the acute phase of intoxication all patients showed eye symptoms of redness, dryness, and irritation; two of them subsequently developed severe bilateral secondary cataracts requiring surgery. A blood test demonstrated the presence of MT-45 at concentrations: 280, 122, and 22 ng/mL (98). However, as the clinical symptoms resembled the toxic actions of chemiotherapeutic agents (98), it is possible that they could indicate the presence of nitrogen mustards, reagents used in the synthesis of MT-45, in poorly purified batches (99).

Twenty-eight deaths associated with MT-45 have been reported by Sweden, all of which were analytically confirmed. These deaths occurred within a short period time, from November 2013 to April 2014; the deceased were male, aged between 19 and 59 years, and a female aged 23 (95). The concentration of MT-45 in postmortem femoral blood ranged from 0.006 to 1.9 µg/g. In 24 of them, MT-45 was found in combination with at least one other psychoactive substance, including anxiolytic/hypnotics (benzodiazepines), antidepressants (fluoxetine, sertraline, mirtazapine, and venlafaxine), antipsychotic drugs (olanzapine, quetiapine, and levomepromazine), antiepileptic drugs (gabapentin, lamotrigine, and carbamazepine), opioids (morphine, codeine, tramadol, fentanyl, and hydrocodone), ethylphenidate, methiopropamine, 2-aminoindane, amphetamine, THC, APBP, and ethanol (95). Very recent case reports of fatal intoxication involving MT-45 (69, 74) are summarized in Table 3.

## The Role of Naloxone in Treating Opioid NPS-Induced Intoxication

Naloxone is a short-acting semisynthetic competitive opioid receptor antagonist with the highest affinity for μ-receptor, though it also blocks δ- and κ-receptors. It is a standard drug for treatment of opioid overdose (100, 101). Naloxone rapidly reverses the clinical signs of opioid overdose, life-treating respiratory depression in particular, and its timely administration is crucial for reducing opioid-linked mortality. Notably, to reduce harms associated with opioids use “… a number of countries have recently adopted policies and procedures that allow medical staff to distribute naloxone to first reponders (e.g., police and firemen) and to people dependent on opioids” (100). In addition to reversing opioid toxidrome, naloxone may induce withdrawal symptoms in a dependent patient who is under the influence of opioids. The drug can be administered by intravenous, intramuscular, subcutaneous, and intranasal routes. The initial dose should be between 0.4 and 2 mg for adults and 0.01 mg/kg body weight in children, given intravenously. If the intravenous route is not available, then intramuscular and subcutaneous administration should be considered. Intranasal delivery may require a higher dose of 4 mg. The dose may be repeated at 2–3-min intervals until the patient is breathing at a rate greater than 10 breaths/minute (100, 101).

## Concluding Remarks

Over the last decade, a significant change has been seen in the use and availability of recreational drugs in various parts of the world, with an increasing number of NPS being observed. While the majority of NPS are designer cannabinoids and psychostimulants, a range of different synthetic opioids have recently appeared on the illicit drug market, namely analogs of fentanyl and compounds with various chemical structures, such as AH-7921, U-47700, and MT-45.

No general population or targeted surveys on the prevalence of illicit use of fentanyls and other novel synthetic opioids were found during this review. Information on the use of these drugs is mostly limited to discussions on user websites. It appears that they are predominantly used by individuals who use illicit opioids, such as heroin and/or prescription opioids, and to a lower extent, by individuals interested in exploring the effects of psychoactive substances, so-called psychonauts. The desired effects are similar to those experienced with heroin: relaxation and euphoria often followed by a sedated, dream-like state.

Vast majority of synthetic opioids was originally synthesized by pharmaceutical companies in a search for effective analgesic drugs with lower adverse effects than morphine. However, due to their toxicity or abuse potential, they were never approved for human medical use. These compounds are opioid receptor agonists which in general are far more potent than morphine. Their effects on humans are largely identical to those of the opioid toxidrome. It should be emphasized that conventional drug tests will not detect synthetic opioids. The growing number of acute intoxication cases, often associated with multidrug abuse, indicates that these drugs should be considered as posing a serious threat to public health. Broad pharmacological, toxicological, and forensic research of these compounds is necessary in order to establish their pharmacokinetic properties, long-term effects, and effective detection methods.

## Author Contributions

JZ prepared and wrote the manuscript.

## Conflict of Interest Statement

The author declares that the research was conducted in the absence of any commercial or financial relationships that could be construed as a potential conflict of interest.

## Footnotes

^1^https://www.bindingdb.org/jsp/dbsearch/PrimarySearch_ki.jsp?energyterm=kJ/mole&tag=lidki&monomerid=50012477&column=KI&startPg=0&Increment=50&submit=Search.

^2^http://drugs.tripsit.me/category/opioid.
